# Translation of a Protease Turnover Assay for Clinical Discrimination of Mucinous Pancreatic Cysts

**DOI:** 10.3390/diagnostics12061343

**Published:** 2022-05-28

**Authors:** Vallabh Suresh, Kaleb Byers, Ummadisetti Chinna Rajesh, Francesco Caiazza, Gina Zhu, Charles S. Craik, Kimberly Kirkwood, Vincent Jo Davisson, Daniel A. Sheik

**Affiliations:** 1 Department of Medicinal Chemistry and Molecular Pharmacology, Purdue University College of Pharmacy, West Lafayette, IN 47907, USA; sureshv@purdue.edu (V.S.); davisson@purdue.edu (V.J.D.); 2 Amplified Sciences, Inc., West Lafayette, IN 47906, USA; kaleb.byers@amplifiedsci.com (K.B.); rajesh.ummadisetti@amplifiedsci.com (U.C.R.); 3 Alaunus Biosciences, Inc., San Francisco, CA 94107, USA; francesco.caiazza@gmail.com; 4 Department of Pharmaceutical Chemistry, University of California, San Francisco, CA 94143, USA; charles.craik@ucsf.edu; 5 Department of Surgery, University of California, San Francisco, CA 94143, USA; gina.zhu@ucsf.edu (G.Z.); kim.kirkwood@ucsf.edu (K.K.)

**Keywords:** pancreatic cancer, early detection, minimal volume, mucinous, non-mucinous, matrix effects, surface-enhanced Raman spectroscopy (SERS)

## Abstract

The classification of pancreatic cyst fluids can provide a basis for the early detection of pancreatic cancer while eliminating unnecessary procedures. A candidate biomarker, gastricsin (pepsin C), was found to be present in potentially malignant mucinous pancreatic cyst fluids. A gastricsin activity assay using a magnetic bead-based platform has been developed using immobilized peptide substrates selective for gastricsin bearing a dimeric rhodamine dye. The unique dye structure allows quantitation of enzyme-cleaved product by both fluorescence and surface enhanced Raman spectroscopy (SERS). The performance of this assay was compared with ELISA assays of pepsinogen C and the standard of care, carcinoembryonic antigen (CEA), in the same clinical sample cohort. A retrospective cohort of mucinous (*n* = 40) and non-mucinous (*n* = 29) classes of pancreatic cyst fluid samples were analyzed using the new protease activity assay. For both assay detection modes, successful differentiation of mucinous and non-mucinous cyst fluid was achieved using 1 µL clinical samples. The activity-based assays in combination with CEA exhibit optimal sensitivity and specificity of 87% and 93%, respectively. The use of this gastricsin activity assay requires a minimal volume of clinical specimen, offers a rapid assay time, and shows improvements in the differentiation of mucinous and non-mucinous cysts using an accurate standardized readout of product formation, all without interfering with the clinical standard of care.

## 1. Introduction

Occurrences of pancreatic cancer are projected to become the second leading cause of cancer-related death by 2030 [1]. Pancreatic cancer has a dismal 5-year survival rate of 11% and its incidence continues to increase [1,2]. This grim outlook arises in part because the disease is most commonly diagnosed at late metastatic stages [3,4,5,6]. Nevertheless, early diagnosis remains difficult because the early stages of the disease are poorly characterized and are largely asymptomatic.

The detection and classification of pancreatic cysts and cystic lesions is an important strategy toward early diagnosis of pancreatic cancer [7]. Pancreatic cysts are incidentally detected in almost half of the patients undergoing magnetic resonance imaging (MRI) and in 2.6% of patients undergoing computed tomography (CT) scans [8,9]. Studies have shown that between 10 and 60 percent of cysts, particularly intraductal papillary mucinous neoplasms (IPMN) and mucinous cystic neoplasms (MCN), have significant potential to develop into malignant cancer [10,11,12,13]. Patients presenting with likely mucinous cysts are candidates for surgical resection, which carries a 2–4% mortality risk, as well as a 21% risk of post-surgical new onset diabetes [14,15]. Disappointingly, surgical interventions are often performed for cysts that are later found to be benign, while patients with non-mucinous cysts could (if accurately classified) be spared costly long-term surveillance [14]. Accurate classification of pancreatic cysts as pre-cancerous mucinous cysts, IPMNs and MCNs, and non-mucinous benign cysts, such as serous cystic neoplasms (SCN), is essential to avoiding misdiagnosis and unwarranted interventions [16,17,18,19].

Biomarker analysis of fine needle aspirate (FNA) fluid collected from pancreatic cysts offers great potential for high accuracy diagnosis of mucinous cysts. The FNA biomarker used most commonly in current clinical assessments is carcinoembryonic antigen (CEA) immunoassay, which has a reported pooled diagnostic sensitivity of 60.4% (95% CI 57.7–62.9) and specificity of 88.6% (95% CI 85.9–90.9) using a 192 ng/mL cutoff level [20]. With room for diagnostic improvement, other biomarkers have also been studied for this purpose. The more intensive analysis of extracted genomic DNA for *KRAS* and *GNAS* mutations shows a pooled 94% sensitivity and specificity of 91% for IPMNs based upon a recent meta-analysis [21]. A recent biomarker discovery effort of cyst fluids identified pepsinogen C (PGC) in mucinous pancreatic cyst [22]. This discovery prompted development of a marker assay based upon the activation of PGC to gastricsin (also known as pepsin C) followed by catalytic turnover of a synthetic substrate tailored for detection by fluorescence resonance energy transfer (FRET) [23]. This assay format was used to discriminate mucinous versus non-mucinous cyst fluid samples from a small retrospective cohort with sensitivity 93%, specificity 100%, and diagnostic accuracy of 95% [22].

The promising results of this previous gastricsin assay is an example of the potential clinical utility of protease activity in cancer diagnostics. As demonstrated for the gastricsin assay, an internally quenched fluorescent substrate offers a general approach to fluorescent signals upon cleavage by target proteases in homogenous solutions such as ADAMTS13 [22,24,25]. A number of additional protease assay platforms have been reported, each with potential utility for clinical applications. Examples including quantum dot-based quenched-fluorescent systems have been successfully used for multiplex protease assays [26]. Nanoparticle-bound fluorescent peptide substrates have been used to measure metalloprotease activity in vivo toward the diagnosis of colorectal cancer [27]. Similarly, substrate-masked antibodies were used to profile metalloprotease activity in cancerous tissues [28]. High sensitivity detection technologies have also been applied to protease activities, including mass spectrometry and surface-enhanced Raman spectroscopy (SERS) [29,30,31,32].

This work demonstrates a gastricsin assay using a magnetic bead-based platform, with both fluorescent and SERS detection modes. The method incorporates a single ultrasensitive dye to enable the detection of low turnover numbers and a highly selective peptide substrate for discrimination of gastricsin activity over other proteases in samples [22,33]. These features have enabled rapid 7 min measurements without significant matrix effects in complex clinical samples, including cyst fluid. Importantly, the concentration of released proteolytic product can easily be assessed, allowing for the quantitative activity analysis rather than the previous FRET cutoff-based readout. Finally, it was demonstrated, using banked cyst fluid samples, that this assay can differentiate mucinous and non-mucinous pancreatic cysts consuming only 1 µL of cyst fluid.

## 2. Materials and Methods

### 2.1. Materials

Synthetic procedures for the dimeric rhodamine 6G dye used in the present study are reported previously [33]. This dye was coupled to the N-terminus of resin-bound biotinylated peptides, purchased from GenScript (Piscataway, NJ, USA). 2-(6-Chloro-1H-benzotriazole-1-yl)-1,1,3,3-tetramethylaminium hexafluorophosphate (HCTU, NC0576737), dichloromethane (DCM, MK-4879-4), trifluoroacetic acid (TFA, AC139720025), diethyl ether (AC12399-0050), acetonitrile for HPLC (A998), and water for HPLC (600-30-13) were all purchased from Fisher Scientific (Pittsburgh, PA, USA). *N*,*N*′-diisopropylethylamine (DIEA, D125806), *N*,*N*′-dimethylformamide (DMF, 319937), hemoglobin (Hb, H7379) and sodium chloride (NaCl, S9625-1KG) were purchased from Sigma Aldrich (St. Louis, MO, USA). Triisopropylsilane (TIPS, A187865) was purchased from Ambeed (Arlington Heights, IL, USA). Tween^®^ 20 (97062-332) was purchased from VWR (Radnor, PA, USA). pH 2 buffer (LC122201) was purchased from LabChem (Zelionople, PA, USA). To prepare gastricsin standards, a recombinant human pepsinogen C (6186-AS-010) was purchased from R&D Systems (Minneapolis, MN, USA). Mock cyst fluid samples were prepared using an artificial mucus matrix, supplemented with pancreatic enzymes, and set to a viscosity of 1.5 centipoise (cP), prepared by Biochemazone (Edmonton, AB, Canada). Streptavidin-blocked sera-mag speed beads (21152104011150) were manufactured by Cytiva (Marlborough, MA, USA). Pepstatin A (S7381) was purchased from Selleckchem (Houston, TX, USA). Trypsin (Research Products International, Mount Prospect, IL, USA, cat. no. T70010-1.0), pepsin (R&D Systems, 6186-AS-010), thrombin (Cayman Chemical Company, Ann Arbor, MI, USA, cat no. 13188), and bovine serum albumin (Sigma Aldrich, A7906-50G) were purchased from their manufacturers. 50 nm citrate-capped silver nanoparticles (1 mg/mL, AGCB50-5M) were purchased from nanoComposix (San Diego, CA, USA). All reagents and proteins required for immunoassay of gastricsin were purchased as part of a kit (DY6186-05) sold by R&D systems. 384 shallow-well, black, flat bottom plate (Thomas Scientific, Swedesboro, NJ, USA, cat. no. 1230M76), and 0.5 mL sterile centrifuge tubes (MTC Bio, Sayreville, NJ, USA, cat. no. C2007) were used. All assay samples were heated on an incubator (VWR, 75838-270), equipped with a 30-tube block (VWR, 13259-000) and a temperature control probe (VWR, 11301-112). All fluorescence readings were measured on a Biotek Synergy H1 Plate Reader. SERS spectra were obtained using a probe-based Raman spectrometer (Wasatch Photonics Inc., Logan, UT, USA model WP-532-SR-IC) with a 532 nm laser source, 50 mW laser power, 25 µm slit width, and standard lenses at 11 mm working distance using 20–30 ms integration time.

### 2.2. Clinical Samples

De-identified clinical samples were obtained under an approved IRB from the UCSF Medical Center and analyzed prior to release of clinical diagnostic information. Samples were banked in collaboration with several medical centers as part of a EDRN study, and no patient eligibility requirements were established for this study to expand the sample pool. The clinical sample diagnoses were established by each institution and clinicians and these data were appended to the sample pool after unblinding along with clinically relevant information including CEA measurements and physical features of the cyst. If clinical information was not available, it was excluded in the data analysis. Briefly, cyst fluid was obtained at the time of operative resection and at the time of endoscopic ultrasound guided (EUS) aspiration. For operative cases, resected specimens underwent cyst aspiration within 30 min of resection. Aspiration was performed by a surgeon, pathologist, or technician. Cyst fluid samples were obtained with an 18-gauge to 21-gauge needle, divided into 100 μL aliquots, and stored at −70 °C or colder. No additives or centrifugation were performed prior to freezing and total time between excision and freezing was <60 min. For EUS obtained samples, aspiration was performed at the time of endoscopy. Cyst fluid was initially allocated for clinically needed tests for care of the patient and any remaining fluid was stored for research purposes. The remaining fluid samples were divided into 100 μL aliquots, and stored at –70 °C. No additives or centrifugation were performed prior to freezing and specimens were frozen within 60 min of aspiration.

### 2.3. Preparation of Assay Buffer

Tween^®^ 20 (0.1%) and NaCl (100 mM) were added to commercial pH 2 Buffer. Assay Buffer preparations were mixed by vortexing and prepared fresh before each assay.

### 2.4. Magnetic Bead Preparation

Synthetic details and characterization of the peptide substrate, VS001 (Figure S1), are given in the Supplementary Information. Streptavidin-coated magnetic beads were loaded with VS001 in bulk. First, an aliquot of beads was twice washed with Assay Buffer, vortexed vigorously, and the beads were separated via magnetic separation with a magnetic rack. The washed beads were then incubated with a volume of 0.05 mM VS001 equal to the starting bead aliquot at room temperature for 15 min with occasional mixing via brief vortexing. After incubation, the beads were washed four times with Assay Buffer using 5× original bead aliquot volume and again isolated via magnetic separation after vigorous vortexing to remove unbound peptide. After the final wash, VS001-loaded beads were resuspended in Assay Buffer to original bead aliquot volume to maintain 10 mg/mL bead concentration.

### 2.5. Assay Buffer Sample Preparation

1 µL recombinant human PGC was diluted with 28.3 µL of Assay Buffer and incubated at room temperature to activate for 10 min. After activation, the 15 µg/mL gastricsin solution was then further diluted to desired concentrations using Assay Buffer prior to protease assay.

### 2.6. Mock Sample Preparation

Mock samples were prepared by diluting 1 µL recombinant human PGC with 28.3 µL Artificial Mucus Matrix to create a 15 µg/mL solution. The mock sample was further diluted with the Artifical Mucus Matrix to desired concentrations, then 1 µL of each Mock Sample was activated by dilution with 99 µL of Assay Buffer followed by gentle mixing and incubation at room temperature for 10 min prior to protease assay.

### 2.7. Clinical Sample Preparation

Clinical samples were thawed on ice and divided into 1 µL aliquots without any additives or centrifugation prior to assay. A single 1 µL aliquot of each sample was diluted with 99 µL of Assay Buffer and gently mixed via pipetting. Samples were then activated for 10 min at room temperature.

### 2.8. Hb Sample Preparation and Assay

Mock samples and Assay Buffer were prepared as described previously. Hemoglobin was hydrated in distilled water before it was spiked to a final concentration of 1.5 mg/mL in an aliquot of freshly prepared Assay Buffer. Mock Samples were run after activation with either standard Assay Buffer or with Assay Buffer supplemented with 1.5 mg/mL of hemoglobin. Whichever buffer was used for activation was used throughout the protease assay and data collection.

### 2.9. General Protease Assay

The protease activity assay was performed in 0.5 mL sterile microtubes where 7.5 µL of VS001 loaded on magnetic beads were added to sample tubes containing 7.5 µL of activated gastricsin sample and gently vortexed before immediate placement on an incubator heated to 37°C for 7 min. After incubation they were promptly placed on a magnetic bead rack to isolate the resulting product solution. Each 15 µL reaction was divided into three 4 µL subsamples pipetted into individual wells of a 384 shallow-well, black, flat bottom plate. Fluorescence intensity was measured in triplicate then the plate was sealed to prevent evaporation prior to SERS analysis.

### 2.10. Protease Comparison Assay

Proteases were hydrated in distilled water according to manufacturers’ specifications before dilution in the Assay buffer. Proteases were activated and assayed according to the General Protease Assay conditions. Reported concentrations are the final concentrations in the assay mixture.

### 2.11. Pepstatin Protease Assay

Two sets of Assay Buffers were prepared as described previously wherein one buffer was spiked with 50 nM of pepstatin A while the other served as a control. Samples containing combinations of 150 ng/mL of gastricsin, and/or 30 nM pepsin in Artificial Mucus Matrix were run normally under General Protease Assay conditions with either Assay Buffer or with Assay Buffer supplemented with 50 nM pepstatin and analyzed concurrently.

### 2.12. Raman Spectroscopy

4 µL of a suspension of silver nanoparticles was added to 4 µL of protease assay sample in a 384 shallow-well plate. The fresh colloid aggregate samples were immediately subjected to Raman spectroscopy to obtain the SERS data. Spectral data were processed and the area under the curve (AUC) was analyzed using OPUS 8.2.28 software (Bruker Optics, Inc., Billerica, MA, USA). Graphs of the data were prepared using GraphPad Prism as described in the Data Analysis section. Error bars represent the standard deviation from triplicate measurements.

### 2.13. Enzyme-Linked Immunosorbent Assay (ELISA)

PGC mass was measured by ELISA according to the manufacturer’s specifications. The total mass of the PGC was measured in all clinical samples using a commercial sandwich ELISA assay for human Pepsinogen C/gastricsin (R&D Systems, Minneapolis, MN, USA), following the manufacturer’s instructions. Optical density was measured with a Biotek Synergy H1 plate reader at 450 nm using wavelength correction at 540 nm. Sample concentration was interpolated from a standard curve of recombinant gastricsin using a 4-parameter logistic regression.

### 2.14. Data Analysis

All data analyses and graph preparations were conducted using GraphPad Prism 9.3. Each sample assay raw fluorescence intensity or SERS AUC was converted to the corresponding product concentration according to the appropriate standard curve as shown in Supplementary Figure S3. Each sample was analyzed in triplicate and the mean and standard deviations were calculated and plotted for each point. For samples with both fluorescence and SERS measurements, paired sample results were plotted against each other to visualize the optical interferences resulting from the external milieu and the linear relationship between the two measurements was calculated. The grey bars in these graphs represent the 95% confidence interval, and the slope is proportional to optical interference of one or both measurements. Unblinded clinical results were plotted according to their diagnosis as either non-mucinous or mucinous, and receiver operating characteristic (ROC) curves were prepared with noted cutoffs, AUC, sensitivities, and specificities. The significance of clinical sample discrimination was analyzed using the Mann–Whitney test for each measurement described, and the *p*-values are listed in the figure caption.

## 3. Results

**Figure 1:**
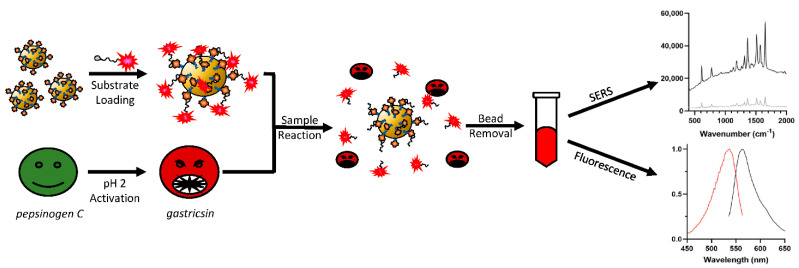
Scheme for conditional gastricsin activity assay. Briefly, magnetic beads are loaded with gastricsin substrate, and any unbound material is washed away. Gastricsin samples (research or clinical) are activated by incubation at pH 2 at room temperature prior to mixing with substrate-loaded beads. Equal volumes of enzyme solution and bead suspension are mixed for a predetermined amount of time, then the beads are removed with a magnetic tube rack. The resulting solution contains enzyme and reaction product at pH 2, which is quantified by either fluorescence or SERS measurements.

The assay design is described in Figure 1. The substrate peptide sequence used was based on previous work [22]. This peptide sequence, selected by multiplex substrate profiling, was shown to be selectively cleaved at low pH by gastricsin without interference from any other proteins found in cyst fluid. To the C-terminus of this peptide, a biotinylated lysine residue was appended and separated from the substrate sequence by a diethylene glycol spacer. At the N-terminus, a dimeric rhodamine 6G (R6G)-based dye was attached, which was previously developed as an ultrasensitive and stable reporter for SERS detection [33]. The resulting substrate was immobilized on magnetic beads coated with streptavidin and used for assaying gastricsin activity. The magnetic beads were loaded with gastricsin substrate, and any unbound material was washed away. Gastricsin samples (standard or clinical) were activated by incubation at pH 2 at room temperature prior to mixing with substrate-loaded beads. Equal volumes of enzyme solution and bead suspension were mixed and incubated at 37°C for 7 min, then the beads were removed with a magnetic tube rack. The resulting solution contained enzyme and reaction product at pH 2, which can be quantified by either fluorescence or SERS measurements.

**Figure 2:**
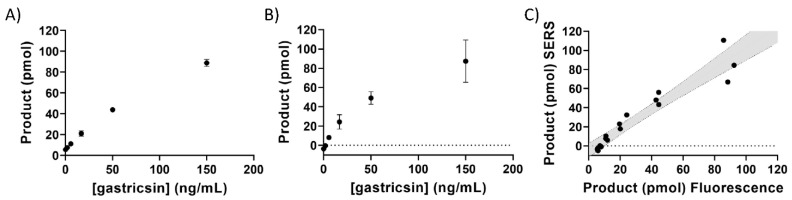
Gastricsin activity assay in pH 2 buffer. (**A**) The product formation is calculated from fluorescence measurement at each concentration of gastricsin as indicated on the x-axis. (**B**) The product formation calculated from SERS measurements at each concentration of gastricsin. The samples in (**A**) were used for these measurements after addition of silver. Error bars represent the standard deviation of triplicate measurements. (**C**) Correlation of product formation between SERS and fluorescence measurements from paired reaction samples. The grey area is the 95% confidence interval from a linear regression analysis with a slope = 1.075 and R^2^ = 0.914.

These assays were responsive to varying amounts of gastricsin (Figure 2). Fluorescence measurements were performed using a 384-well plate in a plate reader. SERS measurements were performed in the same plate after addition of silver nanoparticles. Signals in both detection modes showed a linear dependence with gastricsin concentration in these assays, allowing for direct quantitation of enzymatic activity. Furthermore, results calculated from each of the detection methods correlated well with each other, as evidenced by the linear slope of 1.075 in a plot of SERS vs. fluorescence results, suggesting that both methods were equally well-suited for analysis of gastricsin turnover (Figure 2C). Gastricsin is known to have a broad substrate specificity but cleaves the designed peptide between the alanine and tryptophan residues, as demonstrated previously [22,34]. Using standard curves (Figure S3) made from varying concentrations of a pre-synthesized proteolysis product (Figure S2), the assay was standardized for the product production. As such, it was possible to calculate turnover number and activity in product per unit time.

**Figure 3:**
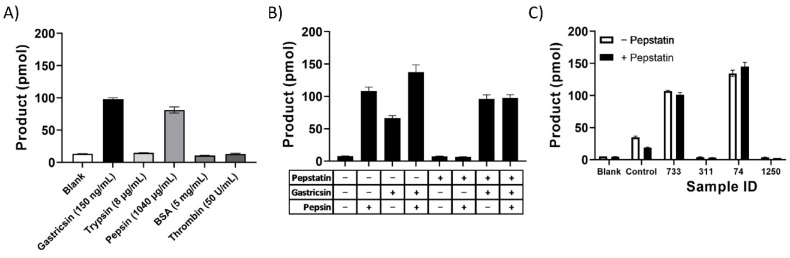
Specificity of assay for gastricsin. (**A**) Activity of VS001 substrate loaded onto beads with proteolytic enzymes and bovine serum albumin (BSA). Concentrations for each analyte are listed in parentheses. (**B**) Impact of 50 nM pepstatin on activity of 150 ng/mL gastricsin and/or 1 µg/mL pepsin in assay as indicated in the table below where “+” indicates addition of analyte. (**C**) Impact of the addition of pepstatin to assay buffer for clinical sample analysis. Controls are mock samples prepared with gastricsin at 150 ng/mL. Error bars represent standard deviation of triplicate measurements.

To ensure the selectivity of this assay for gastricsin, a panel of common proteins and proteases were examined using only the fluorescence readout (Figure 3). While the assay was very selective in this protease panel, some activity was observed with high levels of the structurally similar digestive enzyme pepsin. For the application of classifying pancreatic cysts, this interference is not of concern because pepsin has not been found in cyst fluid to the best of our knowledge [35,36]. However, in matrices containing both gastricsin and pepsin this assay will present limitations. A remedy was to evaluate gastricsin activity in the presence and absence of the selective pepsin inhibitor pepstatin. These results showed that gastricsin was not inhibited by pepstatin, while the activity of pepsin was completely abrogated (Figure 3B). Therefore, in matrices where pepsin is suspected to be present, pepstatin can be used to isolate the signal from gastricsin activity for analysis. This principle was further confirmed by testing several clinical samples activated with and without pepstatin in the pH 2 Assay Buffer. Despite some differences in the positive controls, the clinical samples themselves did not show a change in signal intensity which suggests that these samples did not contain pepsin as expected.

**Figure 4:**
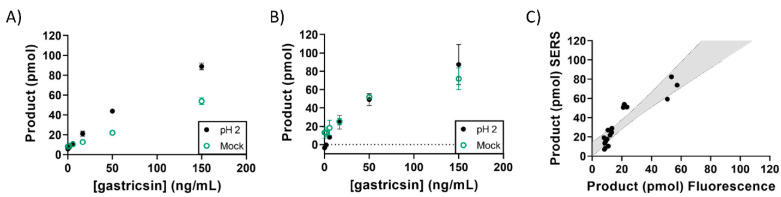
Gastricsin activity assay in samples comprised of a mock mucus matrix containing pancreatic enzymes with a viscosity of 1.5 cP to simulate a pancreatic cyst fluid sample. (**A**) Measured product formation calculated from fluorescence measurement at each concentration of gastricsin as indicated on the x-axis. (**B**) Measured product formation calculated from SERS measurement at each concentration of gastricsin as indicated on the x-axis. The samples are the exact same from panel (**A**). Error bars represent the standard deviation of triplicate measurements. (**C**) Correlation of product formation between SERS and fluorescence measurements from paired reaction samples. The grey area is the 95% confidence interval from a linear regression analysis with a slope = 1.293 and R^2^ = 0.836.

Prior to studies of patient samples, a mock pancreatic cyst fluid preparation was prepared for assay testing and validation (Figure 4). This mixture consisted of an artificial mucus matrix product to which pancreatic enzyme extracts were added. The viscosity of this matrix was adjusted to 1.5 cP, based on previous rheology studies of pancreatic cyst fluid [37]. Recombinant pepsinogen C was diluted with this matrix at varied concentrations and used for gastricsin activity assays. While fluorescence measurements decreased in intensity, SERS analyses showed no matrix effects (Figure 4B). Furthermore, the relationship between product calculated using fluorescence and SERS is skewed, as indicated by the linear slope of 1.293 in Figure 4C. This suggests that, due to decreased fluorescence related to optical interference from the mock matrix, SERS measurements can provide a more accurate analysis of enzyme activity and prove to be more reliable in clinical applications.

**Figure 5:**
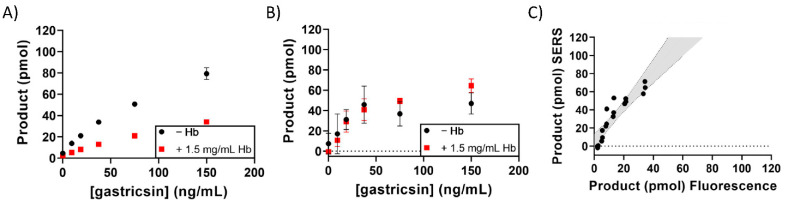
Gastricsin activity assay in samples comprised of a mock mucus matrix containing pancreatic enzymes with a viscosity of 1.5 cP, while the assay buffer contained the indicated concentration of Hb to simulate a bloody pancreatic cyst fluid sample. (**A**) Measured product formation calculated from fluorescence measurement at each concentration of gastricsin as indicated on the x-axis. (**B**) Measured product formation calculated from SERS measurement at each concentration of gastricsin as indicated on the x-axis. The samples are the exact same from panel (**A**). Error bars represent the standard deviation of triplicate measurements. (**C**) Correlation of product formation between SERS and fluorescence measurements from paired reaction samples. The grey area is the 95% confidence interval from a linear regression analysis with slope = 1.940 and R^2^ = 0.828.

Previous reports of cyst fluid sample contamination with blood during fine needle aspiration are of considerable importance to activity assay performance [38]. To anticipate possible optical interferences from hemoglobin in blood-contaminated samples, assays were performed in buffer containing 1.5 mg/mL hemoglobin (Figure 5). The concentration was chosen to model that found in hemolyzed blood [39]. Indeed, fluorescence measurements were decreased in the presence of hemoglobin. Impressively, however, the assay results using SERS-based detection were not compromised. Combined with the studies using mock pancreatic cyst fluid, these data suggest that the SERS-based detection can be used when matrix effects are significant in fluorescence-based activity assays and this hypothesis is supported by the steep slope of 1.940 shown in Figure 5C.

**Figure 6:**
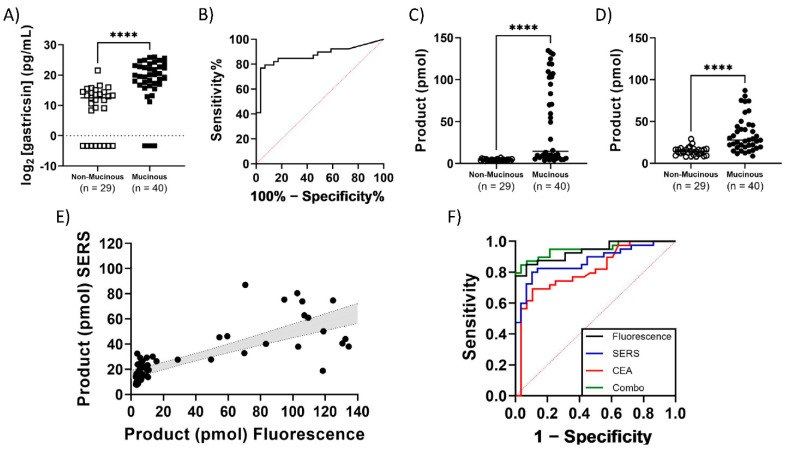
Clinical sample results from a retrospective cohort of 69 pancreatic cyst fluid samples with known clinical diagnoses. (**A**) The measured mass of pepsinogen C according to commercial ELISA kit. (**B**) ROC curve of ELISA measurement of pepsinogen C mass to distinguish mucinous from non-mucinous pancreatic cysts (AUC = 0.873, 95% CI 0.794–0.900). (**C**) Measured product formation calculated from fluorescence measurement of clinical samples. (**D**) Measured product formation calculated from SERS measurement of clinical samples. Sample measurements are paired and both (**C**,**D**) are divided into respective diagnosis category as indicated on the x-axis. (**E**) Correlation of product formation between SERS and fluorescence measurements from paired reaction samples. The grey area is the 95% confidence interval from a linear regression analysis with a slope = 0.348 and R^2^ = 0.614. Every measurement represents a single reaction and sample. (**F**) ROC curve of clinical samples diagnosed according to the gastricsin activity assay using the product formation calculated from fluorescence (black, AUC = 0.936, 95% CI 0.882–0.990) or SERS (blue, AUC = 0.873, 95% CI 0.791–0.956), in comparison with clinical CEA measurement (red, AUC = 0.812, 95% CI 0.707–0.918) and a combination of all three (green, AUC = 0.950, 95% CI 0.900–1.000). **** = *p* < 0.0001 according to a Mann–Whitney test of significance.

Finally, a study of a retrospective cohort of 69 cyst fluid samples with known assignments as mucinous or non-mucinous classification was conducted and optimal cutoff values were established (Table 1, Figure 6). Both fluorescence (AUC = 0.936, 95% CI 0.882–0.990, Sensitivity = 85%, Specificity = 93% at cutoff of 5.79 pmol product) and SERS (AUC = 0.873, 95% CI 0.791–0.956, Sensitivity = 80%, Specificity = 90% at cutoff of 18.72 pmol product) assays were able to differentiate between these two classifications. The assay classifies cysts with better accuracy than the most commonly used biomarker, CEA (AUC = 0.812, 95% CI 0.707–0.918, Sensitivity = 62%, Specificity = 93% at cutoff of 192 ng/mL), as reported in the clinical records for each sample. The accuracy of the activity-based assay was also significantly superior to a protein mass-based pepsinogen C immunoassay (AUC = 0.873, 95% CI 0.784–0.900, Sensitivity = 77%, Specificity = 93% at cutoff of log2 = 16.51 pg/mL). The ROC curve’s AUC is further improved when CEA and gastricsin assays are combined (AUC = 0.950. 95% CI 0.900–1.000, Sensitivity = 87%, Specificity = 93%), suggesting potentially improved diagnostic power when combining biomarkers in diagnosis of cysts. Furthermore, the results for the entire 69 sample cohort were collected and analyzed in a single day, outpacing currently available clinical diagnostics that often require 1–21 days according to clinical laboratory testing criteria available online.

## 4. Discussion

These studies demonstrate a highly accurate magnetic bead-based protease assay platform with both fluorescence and SERS readouts. The work applies the newly developed assay platform to measure gastricsin activity, which has been found enriched in mucinous pancreatic cyst fluids collected through fine needle aspiration, either pre- or post-surgical resection as indicated in Table 1. The gastricsin and CEA analyses can assist clinical decisions regarding the potential risk of cysts to develop into pancreatic cancer. The first step for clinical decision-making is to address whether they are dealing with a benign, non-mucinous, or potentially cancerous, mucinous cyst. Mucinous cysts are significantly more likely to become cancerous but surgical pancreatic resection can prevent this near-universal deadly outcome [10]. As such, accurate diagnosis, aided by the assays, will be useful in determining the risk of the patient developing a pancreatic cancer.

The assay uses a peptide substrate that is selectively cleaved by gastricsin which aims to prevent false negatives. Notably, the presence of pepsin, which is structurally similar to gastricsin, can give a false response in the assays. This situation can be remedied by addition of pepstatin, which selectively inhibits pepsin. While pepsin is not a major concern in cyst fluid applications, this adaptation may be important in other biological matrices. Matrix interference with fluorescence readout in assays are common as also encountered with the gastricsin activity assay in the presence of simulated mucus or hemoglobin. These matrix effects were not a factor in the SERS based readouts. The 69 retrospective cyst fluid samples were quantified using two orthogonal detection methods in the same assay conditions. Impressively, these methods successfully differentiated mucinous and non-mucinous samples.

An important future direction is to study the performance of the assay using larger cohorts of cyst fluid samples since 69 has limited statistical power. The current assay workflow and detection methods offer a robust platform to pursue larger scale clinical studies. Current estimates of sample turnaround times for 100 clinical samples are less than 24 h, even using manual execution. This situation could be greatly improved by adaptation to automated clinical analyzer systems. Technology improvement efforts will seek to incorporate this assay platform onto a system capable of magnetic bead purification and fluorescence readout. Ideally, a system would utilize SERS to remain agnostic to matrix effects. Finally, ultrasensitive SERS-based detection significantly reduces required clinical fluids to nano-sized volumes. Future directions could incorporate nanoliter liquid handling equipment to improve variations. The assay approach offers a suitable platform for improved early detection of pancreatic cancer in conjunction with current clinical standards. Moving forward, this assay will be investigated in an analytical validation study to ensure it is suitable for testing in a CLIA lab setting. Next, the diagnostic accuracy will be evaluated in a clinical validation study prior to release as a lab developed test for discriminating non-mucinous from mucinous pancreatic cysts.

## Figures and Tables

**Table 1:** *n* = 69 clinical sample cohort characteristics. Units are as listed in the row heading.

		Non-Mucinous		Mucinous	
		Pseudocyst *	SCA	MCN	IPMN
**Samples (*n*)**		15	14	13	27
**Age** **(Years ± SD)**		54.2 ± 18.5	60.1 ± 9.6	53.2 ± 17.3	66.8 ± 11.6
**Gender**	Female	7	8	10	13
	Male	7	6	3	14
**Institution**	Pittsburgh	4	0	2	4
	Indiana	6	9	6	13
	Stanford	4	3	5	10
	UCSF	0	2	0	0
Collection Method **	Surgery	6	10	13	24
	EUS-FNA	8	4	0	2
Cyst Size *** **(mm ± SD)**		66.6 ± 33.6	60.2 ± 39.7	54.5 ± 38.1	47.8 ± 32.8
**CEA** **(ng/mL ± SD)**		31 ± 43	7354 ± 27,413	10,773 ± 25,538	5398 ± 16,290
**Gastricsin Mass** **(ng/mL ± SD)**		220 ± 775	10,231 ± 12,485	6972 ± 10,449	10,630 ± 19,431
**Gastricsin Activity (pmol prod ± SD)**	Fluorescence	4.7 ± 1.2	3.9 ± 0.6	56 ± 54	46 ± 47
	SERS	16 ± 5.7	13 ± 3.5	36 ± 22	34 ± 21

* One pseudocyst sample without patient characteristic information. ** One pseudocyst without sample collection information, one IPMN collected via endoscopic retrograde cholangiopancreatography (ERCP). *** Five pseudocyst and two IPMN samples without cyst size data.

## Supplementary Materials

The following supporting information can be downloaded at: https://www.mdpi.com/article/10.3390/diagnostics12061343/s1, Structures and synthetic procedures of VS001 (Figure S1, Figure S4) and gastricsin reaction product (Figure S2, Figure S5) are described and the standard curves of response vs. concentration for the gastricsin reaction product are shown (Figure S3).

## Abbreviations

SERS, surface enhanced Raman spectroscopy; CEA, carcinoembryonic antigen; MRI, magnetic resonance imaging; CT, computed tomography; IPMN, intraductal papillary mucinous neoplasms; MCN, mucinous cystic neoplasms; SCN, serous cystic neoplasms; FNA, fine needle aspirate; PGC, pepsinogen C; FRET, fluorescence resonance energy transfer; cP, centipoise; VS001, gastricsin-specific, dye-labeled substrate; AUC, area under curve; ELISA, enzyme-linked immunosorbent assay; ROC, receiver operating characteristic; R6G, rhodamine 6G.
