# Supplementary material for: Translation of a Protease Turnover Assay for Clinical Discrimination of Mucinous Pancreatic Cysts
Source: Diagnostics (Basel). 2022 May 28;12(6):1343. doi: 10.3390/diagnostics12061343 (PMC9222202; doi:10.3390/diagnostics12061343)
Supplement: Supplementary file 1 [file diagnostics-12-01343-s001.zip › diagnostics-1737707-supplementary.pdf]

SUPPLEMENTAL MATERIAL

Translation of a Protease Turnover Assay for Clinical Discrimination of Mucinous Pancreatic Cysts

Vallabh Suresh[1,†], Kaleb Byers[2,†], Ummadisetti Chinna Rajesh[2], Francesco Caiazza[3,4], Charles Craik[4], Kimberly Kirkwood[5], Vincent Jo Davisson[1], Daniel A. Sheik[2]*

[1]Department of Medicinal Chemistry and Molecular Pharmacology, Purdue University College of Pharmacy, West Lafayette, IN 47907

[2]Amplified Sciences, Inc, West Lafayette, IN 47906

[3]Alaunus Biosciences, Inc, San Francisco, CA

[4]Department of Pharmaceutical Chemistry, University of California San Francisco,

[5]Department of Surgery, University of California, San Francisco, CA

*Address correspondence to this author at: Amplified Sciences, 1281 Win Hentschel Blvd., Suite 2132, West Lafayette, IN 47906. Email: Dan.Sheik@amplifiedsci.com

† These authors contributed equally to this work

Table of Contents

1) **Figure S1. Structure and Synthesis of VS001** **Page 2**
2) **Figure S2. Structure and Synthesis of Dye Labeled Gastricsin Product** **Page 3**
3) **Figure S3. Standard Curves of Product** **Page 4**
4) **Figure S4. VS001 Purity** **Page 5**
5) **Figure S5. Dye-labeled Gastricsin Product Purity** **Page 6**

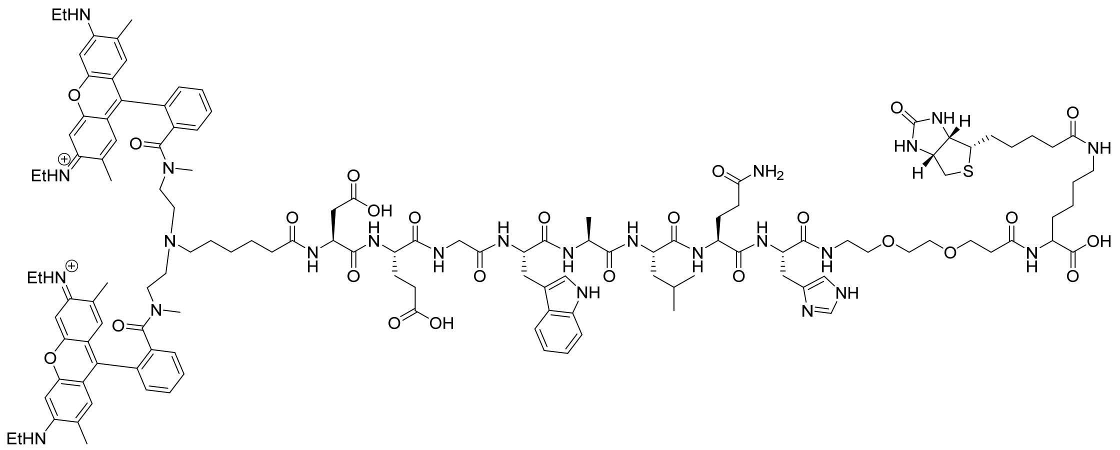

Supplemental Figure S1. Structure of peptide substrate, VS001.

(1) Synthesis and Characterization of Dye-labeled Peptide Substrate, VS001

To 13 µmol of resin-bound peptide substrates, a preactivated mixture of dimeric rhodamine 6G dye (1.1 eq, 14.8 µmol, 16 mg), HCTU (1.1 eq., 14.8 µmol, 6 mg), and DIEA (3.3 eq., 44.4 µmol, 8 µL), in 2 mL of DMF was added. This mixture was incubated at room temperature for 18 hours with mechanical shaking. The resin was then filtered and washed with DMF (6x) and DCM (3x) sequentially. The peptide substrate, **VS001**, was then cleaved from the resin by addition of 10 mL of TFA:TIPS:Water (95:2.5:2.5) mixture and shaking for 2 hours. The cleavage solution was isolated by filtration and the crude dye-labeled peptide (**VS001**) was concentrated in vacuo, precipitated with diethyl ether, and purified by high performance liquid chromatography (HPLC).

For semi-preparative separations, an Alltech Econosil C18 column was used. The compound was purified by eluting with water and acetonitrile, each containing 0.01% trifluoroacetic acid (solvent A and solvent B respectively). A gradient elution, with a constant flow rate of 3 mL, was performed: 5% solvent B in A for the first 5 minutes, followed by a linear increase from 5% solvent B to 95% solvent B from until 20 min., a wash period of 95% solvent B from 20 to 25 min., and finally a linear decrease from 95% to 5% solvent B in A from 25 to 30 min. All material absorbing at 520 nm was collected, combined, and dried. The product was confirmed by electrospray mass spectrometry, performed using an Advion Expression^{L} spectrometer (**M^{2+}:** Calc: 1244.64, Found: 1244.4; **MH^{3+}:** Calc: 830.10, Found: 830.1).

For purity analysis, a Vydac Protein and Peptides C18 column was used. A gradient elution, with a constant flow rate of 1 mL, was performed: 5% solvent B in A for the first 5 minutes, followed by a linear increase from 5% solvent B to 95% solvent B from until 20 min., a wash period of 95% solvent B from 20 to 25 min., and finally a linear decrease from 95% to 5% solvent B in A from 25 to 30 min. The compound was eluted at 16.09 as a peak containing a shoulder feature, observed at multiple wavelengths using a Hitachi L7450 detector. When this shoulder was collected and analyzed again, the same peak with a shoulder feature was observed, indicating that this behavior

was not due to an overlapping impurity. Detection at 220 nm showed an impurity at 15.07 min., which was not observed at 520 nm. Analysis of peak areas determined that VS001 was 96% pure.

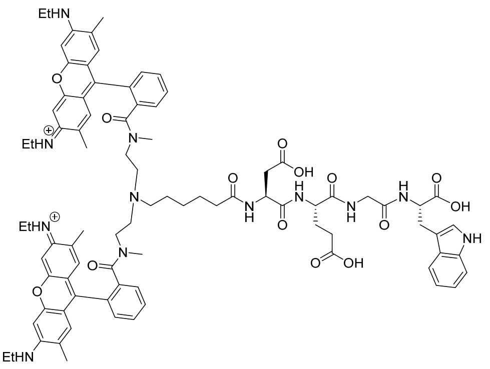

Supplemental Figure S2. Structure of dye-labeled gastricsin product.

(2) Synthesis and Characterization of Dye Labeled Gastricsin Product

Synthesized gastricsin product was prepared analogously to **VS001** (see previous procedure). The resin-bound peptide sequence was ordered from genscript, labeled with dimeric rhodamine dye, cleaved from resin, purified using semi-preparative HPLC on an Alltech Econosil Column (using the same method as described for **VS001**), and concentrated in vacuo. The peptide was identified using electrospray ionization mass spectrometry, performed using an Advion Expression^{L} spectrometer (**M^{2+}:** Calc: 763.88, Found: 764.0).

For purity analysis, a Vydac Protein and Peptides C18 column was used. A gradient elution, with a constant flow rate of 1 mL, was performed: 5% solvent B in A for the first 5 minutes, followed by a linear increase from 5% solvent B to 95% solvent B from until 20 min., a wash period of 95% solvent B from 20 to 25 min., and finally a linear decrease from 95% to 5% solvent B in A from 25 to 30 min. The compound was eluted at 17.27 min., as a single sharp peak, by detection using UV-visible absorption at 220 nm (using a Hitachi L7400 detector). Because no other peaks were observed, the product was deemed to be greater than 99% pure.

(3) Standard Curves of Product

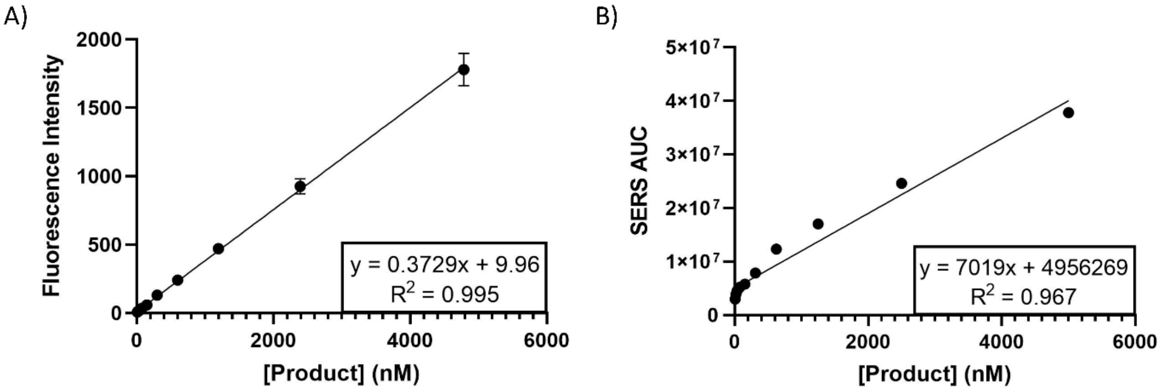

Supplemental Figure S3. Standard curves using fluorescence intensity (**A**) or SERS AUC (**B**) measurements of the product of the enzyme reaction at the concentrations listed on the x-axis. Samples were run in triplicate for fluorescence and a single well of each sample was run using SERS. The curves were fit with a linear regression and the equation and fit are shown in each graph.

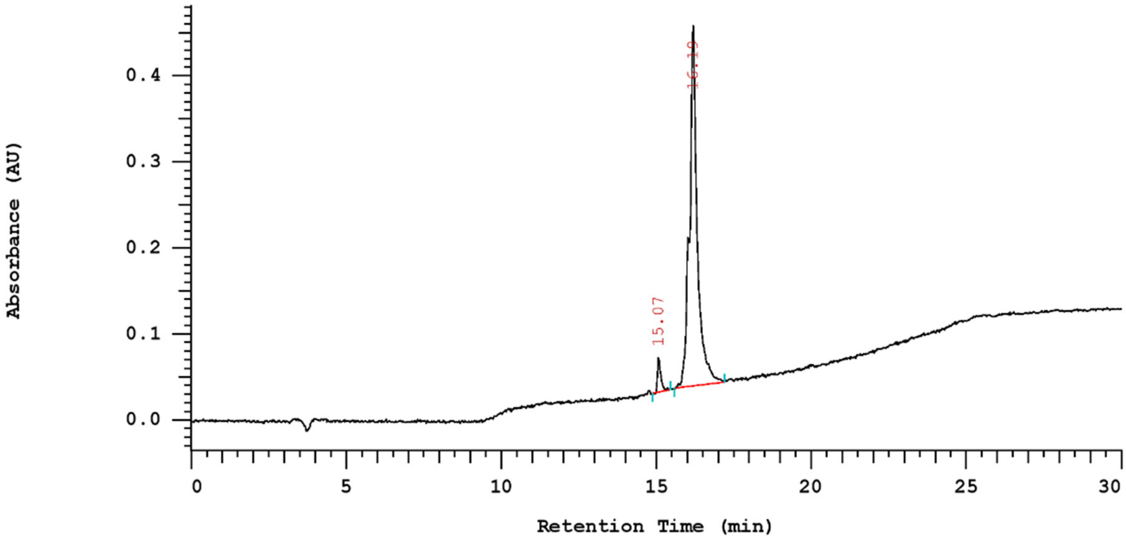

Retention Time	Peak Area	Percentage
15.07 min.	145480	3.83%
16.19 min.	3650080	96.167%

Supplemental Figure S4. Chromatogram and purity of VS001.

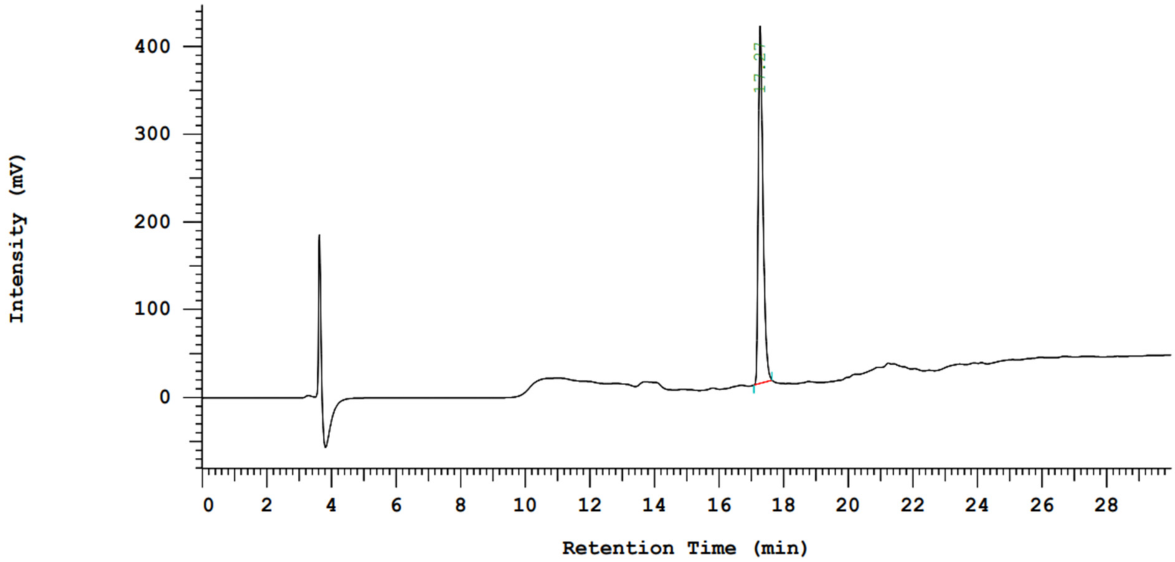

Supplemental Figure S5. Chromatogram and purity of VS001.
